# Mortality rate of Boer, Central Highland goat and their crosses in Ethiopia: Nonparametric survival analysis and piecewise exponential model

**DOI:** 10.1002/vms3.876

**Published:** 2022-07-10

**Authors:** Erdachew Yitagesu, Enyiew Alemnew

**Affiliations:** ^1^ Debre Birhan Agricultural Research Center Debre Birhan Ethiopia; ^2^ College of Veterinary Medicine and Animal Sciences Department of Veterinary Epidemiology and Public Health University of Gondar Gondar Ethiopia

**Keywords:** Boer, Central Highland goat, mortality, nonparametric survival analysis, piecewise exponential model

## Abstract

**Background:**

Analysis of survival data by linear or logistic model ignores censoring and skewness inherent in the mortality data. The objective of this study is to estimate the morbidity and mortality rates of kids and adult goats, identify important risk factors for mortality using survival analysis and summarise important causes of goat death.

**Methods:**

Nonparametric survival analysis and a piecewise exponential model (PEM) were used.

**Results:**

The mortality rates of kids and adult goats were 0.629 and 0.302 per animal year, respectively. The 25th, 50th and 75th percentiles of survival time of kids were 5, 162 and 1300 days, respectively, and those of adults and goats were 280, 828 and 1,735 days, respectively. Gastrointestinal‐related diseases, pneumonia, weak kid, agalactia, mismothering and heartwater (cowdriosis) were the most important causes of mortality. Constant piecewise exponential regression analysis of risk factors indicated that breed, kid birth weight, doe post‐partum weight (PPWT), birth type, birth year and precipitation variables were associated with (*p*‐value < 0.05) kid mortality rate. Pure Boer kids compared with Central Highland goat cross with Boer goats were 2.505 times at a higher probability of mortality (*p*‐value < 0.001). A 1‐kg increase in kid birth weight and dam PPWT reduces mortality probability by 32.5% (*p*‐value = 0.000) and 6.4% (*p*‐value < 0.001), respectively. Twin birth kids had a 1.512 times higher rate of mortality (*p*‐value = 0.001) than single‐born kids. A 1‐ml increment of 15 days of average precipitation significantly reduced kid mortality by 7.8% (*p*‐value < 0.001).

**Conclusion:**

Vaccination, extensive control of ticks and the use of proper comfortable housing to reduce the stress of goats are recommended. Scheduling a mating programme (May to early July) to match the kidding period to the less kid mortality seasons (end of the long rainy season) of flocks is also important to reduce kid mortality.

## Graphical abstract

The hazard of mortality is higher in the early age of kids and at the early time of goats that joined the farm. Both kid birth weight and doe post‐partum weight was negatively associated with the hazard of kid mortality. Increased environmental precipitation reduces the hazard of kid mortality.

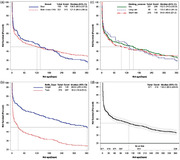

## 1 INTRODUCTION

The goat population of Ethiopia is estimated to be more than 32.74 million (CSA, 2017). In Ethiopia, there were attempts in the early 1970s to cross Saanen with Afar and Highland goat types and between 1989 and 1997 to cross Anglo‐Nubian with indigenous Somali goat breeds to enhance the productivity of indigenous goats. However, both programmes were not sustainable since the effort was not supported by appropriate extension packages, including health, feed and management. In addition, the crossbreeds did not generate more net benefit than the local breeds (Ayalew et al., 2003; Yami & Merkel, 2008). Virtually, all small ruminant (sheep and goats) crossbreeding programmes in the tropics were not successful. This is due to the incompatibility of the genotypes with the breeding objectives, management approaches of the prevailing low input production systems of the area, absence of involvement of livestock owners and stakeholders in decision‐making and ownership of the initiatives or low regard to the potential of indigenous breeds (Abraham et al., 2019).

The introduction of the Boer goat breed to Ethiopia was started by inseminating Arsi‐Bale goat ewes with Boer goat semen at Hawassa University, and Somali ewes are also inseminated at Haramaya University to produce F 1 crosses; the preliminary results were promising (Yami & Merkel, 2008). Boar goats, as an improver to local goats for meat production, have been imported, and breeding work is ongoing (Molla, 2016; Tesema et al., 2017; Mustefa, Gizaw, et al., 2019). Debre Birhan Agricultural Research Center, at Ataye Boer goat evaluation research site, started cross breeding and evaluation of Boer goat with Central Highland goat (CHG) in 2011 by importing Boer goats from the Republic of South Africa and purchasing CHG ewes from nearby farmers. The evaluation was continued up to the end of 2018. The reproductive performance of ewes, kid survival rate and mortality statistics were published from this evaluation research (Alemnew, Yitagesu, et al., 2020; Mustefa, Gizaw, et al., 2019; Mustefa, Banerjee, et al., 2019). However, there were gaps in incorporating important risk factors for kid and adult goat mortality, performing statistical assumptions and using important statistical models for survival analysis and did not include all goats and their life time period on the farm.

The mortality data constitute a special category of data known as time‐to‐event data, characterised by whether and when an event occurs during a study period. Mortality data are skewed and are subject to ‘censoring’, which occurs when the information about survival times of some individuals is incomplete. The most common form of censoring is ‘right censoring’, in which an event of interest (e.g., mortality) for the duration of the study is not observed. The survival data can be analysed by linear and logistic regression methods. Analysis of survival by linear model ignores censoring and skewness inherent in the mortality data (Ellen et al., 2010). Moreover, considering mortality as a binary trait causes severe information loss because animals dying early or late in the study period cannot be differentiated. The piecewise exponential model (PEM) is an extension of the exponential proportional hazards model used in modelling time‐to‐event data. It appears to be more flexible than the popular standard Cox model in terms of hypothesis testing. Another advantage of the PEM over the Cox model is that it is possible to compute the hazard rate within each interval. Given a series of time intervals, the baseline hazards are known to be constant within each interval but not necessarily constant across the different intervals defined by the change points (Allison, 2010). The objective of this study is to estimate the morbidity and mortality rates of kids and adult goats, identify important risk factors for mortality and summarise important causes of goat death during the study period.

## 2 MATERIALS AND METHODS

### 2.1 Study area and flock management

**FIGURE 1:**
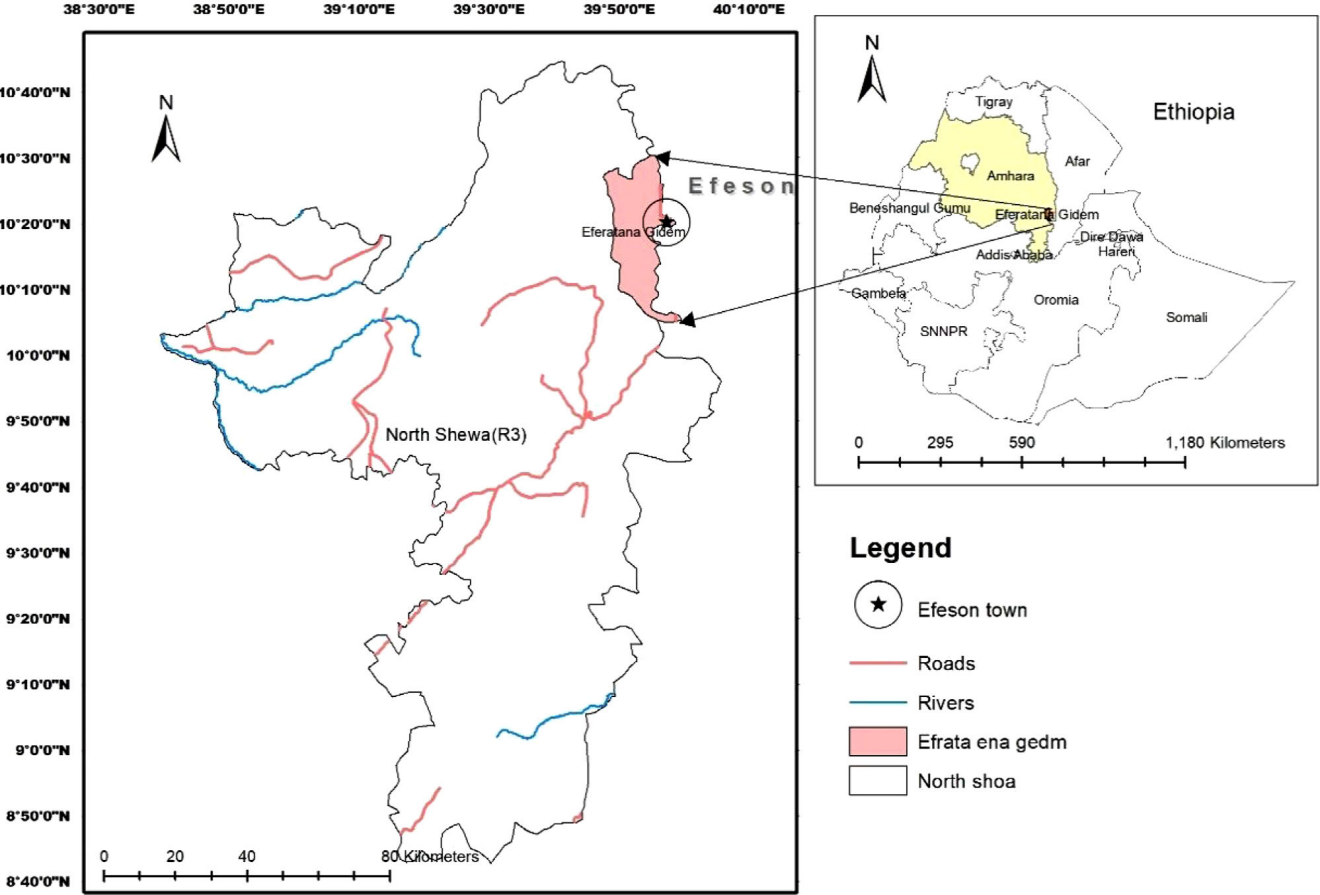
Map of Ethiopia showing the region, zone, district and town where the study site is located

The study was conducted at the on‐station Boer x CHG cross‐breeding programme of Debre Birhan Agricultural Research Center, at Ataye (Efratana Gidim district) research site, Ethiopia. In general, three seasons exist in Ethiopia: (i) the main rainy season (June–September, called Kiremt), (ii) the short rainy season (March–May, called Belg) and the dry season (October–February, known as Bega). Kiremt rainfall contributes the most to the annual rainfall total and covers most parts of the country except the south and southeast areas (Seleshi & Zanke, 2004). The Efratana Gidim district is located in the lowland agro‐ecological zones of central Ethiopia, and the climate is characterised by bimodal rainfall and consists of a long rainy season (June–September), a short rain season (February–May) and a dry season (October–January) (Alemayehu & Bewket, 2017; Fekadu, 2015). The Efratana Gidim district receives an annual rainfall of approximately 1013.6 mm, with 65.8%, 20.6% and 13.6% contributed by the Kiremt, Belg and Bega seasons, respectively. The average seasonal temperature ranges from a minimum of 11.3°C in the Bega season to a maximum of 31°C in the Kiremt season (Alemayehu & Bewket, 2017). The site's geographic coordinate reference is 10035′ N latitude, 390 93′ E longitude and 1491 m above sea level altitude (Figure 1). Geographic coordinate references of the Efratana Gidim district in its region and zone are displayed in Figure 1.

The goat flock was a mix of Boer, Boer cross with CHG and CHG breeds. The research site started cross‐breeding and evaluating by cross‐breeding Boer goat with CHG in 2011 by importing Boer goats from the Republic of South Africa and quarantined at Sebeta before being transported to the research station. The CHG ewes were bought from the local market available around the site. The evaluation was continued up to the end of 2018. The flock was managed under a semi‐intensive production system with grazing and supplement. The supplement includes ad libitum grass hay, chopped pasture (Napier grass, *Desmodium* spp. and vetch) and commercially prepared concentrate mix (wheat bran and noug seed cake [67:33% ratio]); 300–500 g/head/day to the adults and 100–200 g/head/day to the kids based on their body weight. The pasture feed given to the flock depends on the forage availability across the year in the forage land. The flock health management was maintained through regular follow‐up and treatment of clinical cases. Regular deworming (during short and long rainy seasons) using Albendazole, Tetraclozan, Levamisole, Fascinex and Ivermectine 1% based on coprological examination for internal parasite infestation; regular spraying for ticks, mites and other ectoparasites with Diazinon 60% and vaccination for major bacterial and viral small ruminant diseases (sheep and goat pox, ovine pasteurellosis, pestides petitis ruminantis [PPR] and contagious caprine pleuropneumonia) in the area were done. A detailed summary of the breeding, feeding, management system and data recording of the flock are presented in Mustefa, Gizaw, et al. (2019).

### 2.2 Study design

#### 2.2.1 Descriptive, nonparametric survival analysis and PEM

Data related to mortality collected during the follow‐up period were entered into an Excel spreadsheet. The time goats entered the farm through purchase, transfer or by birth was the starting point, and the time of death was the failure time. Daily precipitation data were taken from the near‐farm Majete climate data collection substation. Fifteen days of average precipitation were calculated and recorded for kids at their birth date. Censored observations are goats that leave the farm by transfer or the end of the study period. All analyses were performed using SAS statistical software version 9.4 (SAS Institute, 2015) and STATA software version 16 (StataCorp, 2019). Estimation of the survivor function was computed as follows (Kaplan & Meier, 1958).

(1) $$\hat{S}\left(t\right)=\prod {}_{j:t_{j}\leq t}\left(1-\frac{d_{j}}{n_{j}}\right),$$

where *Ŝ* (*t*) is the value of the survival function at time *t_j_*, *n_j_* is the number of dead goats at time *t_j_* and *d_j_* is the number of dead goats at time *t_j_*. Survival curves were constructed with the Kaplan–Meier method, and we used the % NEWSURV survival curve plotting macro (Meyers, 2017).

The PEM is a survival model in which the time scale is divided into intervals, and the hazard function is assumed constant within each interval (Allison, 2010). If there are *L* periods, the piecewise constant transition rate is defined by *L* parameters. The central idea of the piecewise model is that only a baseline rate, given by period‐specific constants, can vary across periods but that the covariates have the same (proportional) effects in each period. We install a STATA ado‐file (stsplit) that will automatically split the episodes and estimate the piecewise constant exponential model (Cleves, 2010). Similar to the Cox proportional hazards model, PEMs model the conditional hazard function using a proportional hazard framework with a constant but different baseline hazard within a priori defined intervals. The time‐varying effects weaken the proportional hazards assumption from ‘same effect over entire follow‐up’ to ‘same effect within an interval of follow‐up’ which should better approximate the nonproportional hazards inpatient mortality after listing (Blackstone et al., 2018). We used time points at 7, 90 and 180 days to split the overall time period into four episodes:

(2) $$h\;\left(t|x\right)=c_{k\;}\;\;x\;\exp\left\{X^{T}\;B+\;X^{T\;}B_{k}\;\right\},\;\mathrm{when}\;t\;\in \left(I_{k-1\;},\;I_{k}\right),$$

where *c_k_* is the baseline hazard for interval *k*, *I _k_* for *k* = 0,…,*m* are the partition points that define each interval, *β* is the overall covariate effect and is constant over time, and *β_k_* is the deviation of the covariate effect for interval *k* from the overall effect.

## 3 RESULTS AND DISCUSSION

### 3.1 Descriptive statistics and nonparametric survival analysis of morbidity and mortality of goats

From 671 kids born during the follow‐up period of 2011/2012–2018/2019, a mortality rate of 464 (464/671 = 69%, 95% confidence interval [CI]: 64%–73%) was recorded. Similarly, from 347 adult goats that joined the farm and were followed during the follow‐up period, a mortality rate of 252 (252/347 = 73%, 95% CI: 67.61%–77.25%) (cumulative mortality) was recorded. The mortality rates of kids and adult goats were 0.629 and 0.302 per animal year, respectively. The 25th, 50th and 75th percentile survival times of kids were 5, 162 and 1300 days, respectively, and those of adult (yearling) goats were 280, 828 and 1735 days, respectively. Incidence rate reports are better than prevalence reports for the accurate comparison of epidemiological reports; however, incidence rate reports are very few in animal health studies.

Kid mortality is a major constraint for improving the efficiency of small ruminant production systems in the dry tropics (Hary, 2002, 2000; Gemiyu, 2009; Molla, 2016; Tesema, Alemayehu, et al., 2020; Zeleke, 2007). The present mortality rate is relatively higher than reports of Boer cross‐breeding and evaluation research sites in Ethiopia as well as abroad. The 25th and 50th percentile survival times of 5 and 162 days (∼5 months) in kid in the present study were shorter than 22.2%, 33.8% and 42.1% of the failure rates at 3, 6 and 12 months of kid age, respectively, and 6.73% and 16.6% mortality rates at pre‐weaning and post‐weaning age , respectively (Belay et al., 2014; Tesema, Alemayehu, et al., 2020). At the Jinka Agricultural Research Station, a 45% pre‐weaning mortality rate was reported, which is closer to our report (Molla, 2016). Similarly, more than half (56.7%) of the kid crops died within 4 months in the small rainy season period (March to June) in Alaba Special District, South Ethiopia (Gemiyu, 2009). Kid mortality rate was rapid in the first few weeks of their age (5 days 25th percentile survival time). Similarly, adult goats were at higher risk of mortality in the first few months (∼9 months) (280 days 25th percentile survival time) after joining the farm. The mortality rate was higher in newborn kids than in adult (yearling) age goats (0.629 vs. 0.302 mortality rate per animal year). Higher mortality rate trends of kid and lamb at their early age were reported in Ethiopia (Getachew et al., 2015; Tesema, Alemayehu, et al., 2020; Tesema, Deribe, et al., 2020). Our result is in line with 22.3% of kid loss within 48 h reported in South Africa (Lehloenya et al., 2005). The mortality rate was higher in Boer goat breeds in both age groups. Lehloenya et al. (2005) also reported a higher mortality rate of the Boer goat breed kid than the Nguni goat breed kid. The mortality rate was higher in Boer goat breeds in both age groups. The higher mortality rate of goats during the early time in the farm may be due to adaptation failure of goats in the new environment and the management system of the area (Table 1).

**TABLE 1:** Descriptive statistics and mortality rate of goats at Ataye Boer centre (N = 1018)

						Percentile survival time (days)		
Age	Breed	Time at risk (animal years)	IR	All goats	Died	25%	50%	75%
Newborn (kids)	Boer	129.15	0.836	150	108	8	199	841
	CHG cross Boer	593.83	0.584	521	347	4	153	1955
	Subtotal	722.98	0.629	671	464	5	162	1300
Adult (yearling)	Boer	398.39	0.306	142	122	513	796	1653
	CHG	434.88	0.299	205	130	64	980	2044
	Subtotal	833.27	0.302	347	252	280	828	1735
Total		1556.25	0.454	1018	707	24	472	1568

Abbreviations: CHG, Central Highland goat; IR, incidence rate per animal year.

The distribution of causes of goat mortality and their relative contributions are presented in Table 2. During the study period, many disease syndromes were diagnosed through ante‐mortem and post‐mortem clinical diagnosis methods. Most of the causes of death were unknown (no clear ante‐mortem and/or post‐mortem lesion) (44.50%). Gastrointestinal‐related diseases (diarrhoea, internal parasites and others), pneumonia, weak kid, agalactia and mismothering (also called starvation‐mismothering exposure complex), heart water (cowdriosis) and others were the most common disease syndromes diagnosed as causes of goat mortality on the farm (Table 2). Gastrointestinal‐related diseases (internal parasites, diarrhoea) and pneumonia are the most important causes of kid mortality (Mukasa‐Mugerwa et al., 2000; Donkin & Boyazoglu, 2004; Tesema, Alemayehu, et al., 2020; Tesema, Deribe, et al., 2020). Most proportions of kids died due to agalactia and mismothering (starvation‐mismothering complex [SME]), weak kid and unthriftiness. These problems are correlated with one another. Hary (2000) also reported that in most causes of kid mortality (38% of all observed kid losses), there were no clear diagnosis established. Often, these events are thought to be related to the so‐called SME complex. Dystocia or stillbirth (including birth injury) and the SME complex are the predominant causes of death in Australian lambs and kids and are responsible for ∼80% of perinatal deaths (Robertson et al., 2020).

**TABLE 2:** Causes of goat mortality and their relative contributions at Ataye Boer centre

Cause of death	Newborn	Adult (yearling)	Total	Relative percentage
Unknown causes	213	95	308	43.50
GIT problems vs. internal parasite	25	48	73	10.31
Pneumonia	40	24	64	9.04
Miss‐mothering and agalagctia	59	0	59	8.33
Weak kid	37	0	37	5.23
Heart water (cowdriosis)	18	15	33	4.66
Systemic infection	8	14	22	3.11
Internal parasite	15	6	21	2.97
External wound	13	6	19	2.68
Dystocia	4	12	16	2.26
Unthriftiness	16	0	16	2.26
Liver fluke	2	12	14	1.98
Caseous lymphadenitis	3	8	11	1.55
Sudden death	2	10	11	1.55
Aging	0	2	2	0.28
Predator	0	1	1	0.14
Total	455	253	708	100

Abbreviation: GIT, Gastrointestinal.

During the follow‐up period, most of the clinical diseases were diagnosed related to the integumentary system (skin abscess, caseous lymphadenitis), respiratory system diseases (pneumonia), gastrointestinal‐related disorders (diarrhoea) and others (Table 3). In line with our results, respiratory problems, gastrointestinal parasites and local skin abscesses were reported in recently imported Boer goats breeding and evaluation research centres (Asres et al., 2014; Hunduma et al., 2010; Molla, 2016).

**TABLE 3:** Most frequently diagnosed goat diseases category and their relative contribution at Ataye Boer centre

Disease category	Frequency	Relative percentage
Integumentary	509	25
Respiratory	429	21
Gastrointestinal	333	16
Reproductive	238	12
Nervous	217	11
Metabolic	126	6
Other infectious	124	6
Musculoskeletal	52	3
Total disease cases	2028	100

**FIGURE 2:**
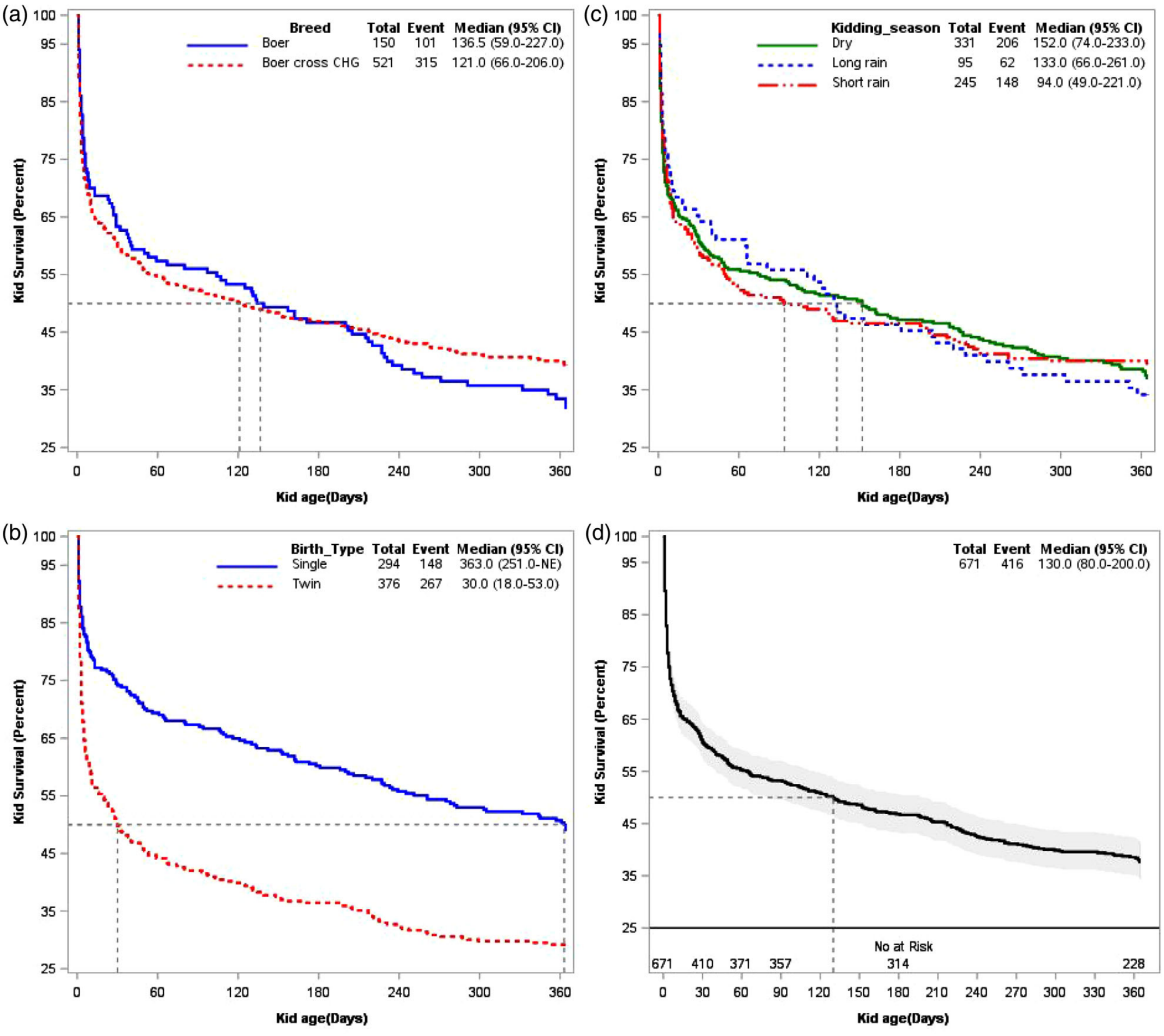
Kaplan–Meier survival function curve of kid mortality (671) and risk factors from birth to 1 year of follow‐up at the Ataye Boer centre

The unadjusted Kaplan–Meier survival function curve from birth to 365 days of follow‐up period of kid mortality stratified based on breed of goat, season and birth type at kid birth indicates that the mortality rate is steady and the overall median survival time is approximately 130 days (Figure 2d). The mortality rate of pure Boer breed kid is lower at an early age than that of CHG cross Boer kids; however, the mortality rate of the Boer goat breed is higher after approximately 190 days of kid age (Figure 2a). The hazard of mortality is higher for twin birth kids than for single birth kids (Figure 2b). Even though the effect of season is not constant overtime, kids born during the short rain season are at higher risk of mortality (shorter median value, 94 days) (Figure 2c).

**FIGURE 3:**
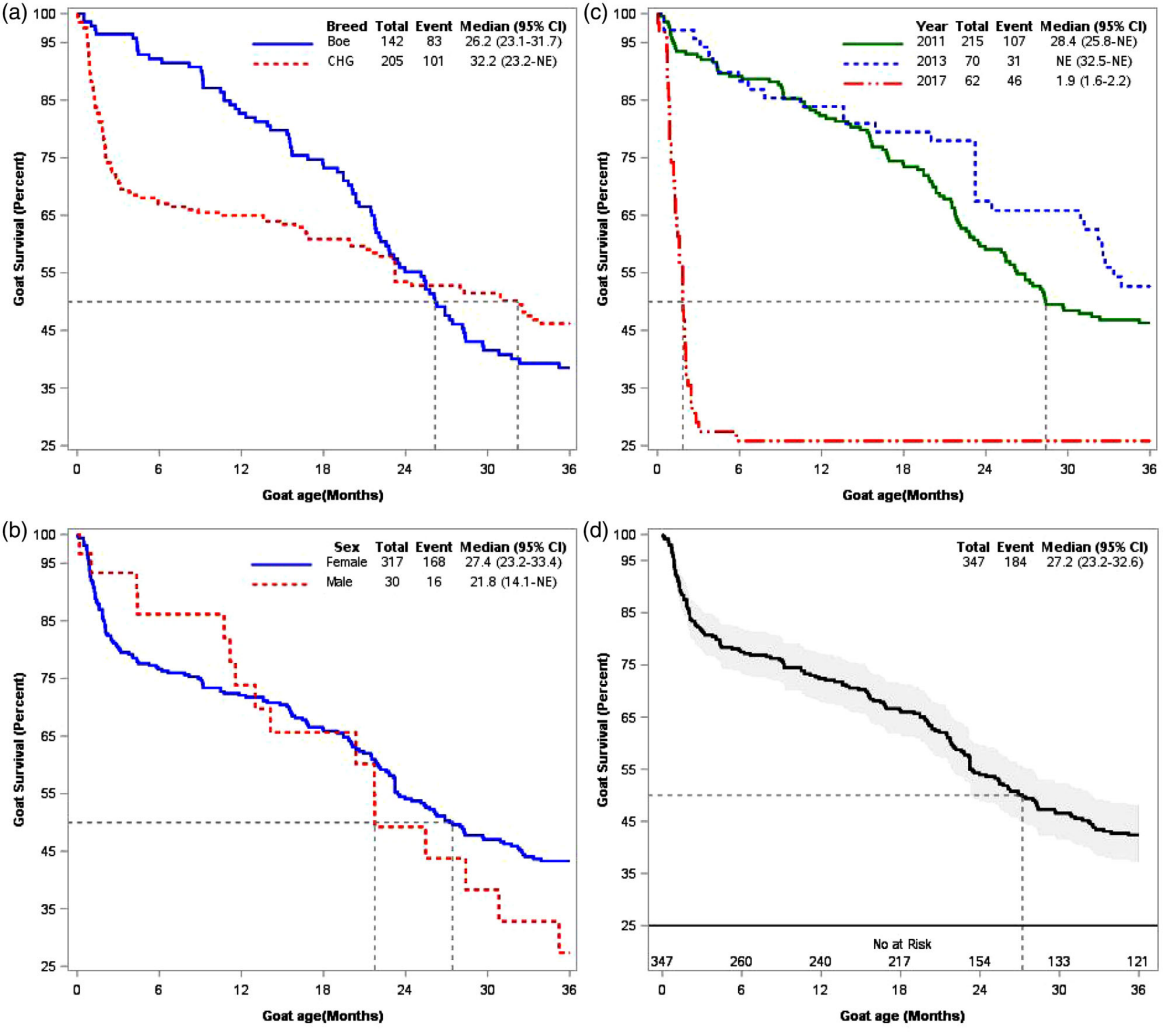
Kaplan–Meier survival function curve of yearling age goat mortality (347) and risk factors from entrance to farm to 3 years of follow‐up at the Ataye Boer centre

The unadjusted Kaplan–Meier survival function curve from the entrance to the farm (at their yearling age) to 36 months of the follow‐up period of mortality stratified based on breed and sex of goat and year of entrance to the farm indicates that the mortality rate is steady and the overall median survival time is approximately 27.2 months (Figure 3d). The mortality rate of the CHG breed is higher than that of pure Boer (Figure 3a), and goats that joined in 2017 had a higher mortality rate (Figure 3c). Goats that joined the farm in 2017 died due to the PPR outbreak. This may be because the goats purchased from different farmers were not previously vaccinated, and some of the goats were infected prior to joining the farm (Alemnew, Yitagesu, et al., 2020). The effect of sex varied over time. Females were at higher risk of mortality at early time, while males were at higher risk of mortality at the late time since they joined the farm. However, the proportion of males was lower than that of females (Figure 3b).

### 3.2 Piecewise exponential model

Constant piecewise exponential regression analysis of risk factors indicated that breed, kid birth weight, doe PPWT, birth type, birth year and precipitation variables were significantly associated with (*p*‐value < 0.05) kid mortality rate. However, kid sex and doe parity number were not significantly associated (*p*‐value > 0.05) with kid mortality rate. Boer‐breed kids had a 2.505 times higher probability of mortality than CHG cross Boer goat kids (*p*‐value < 0.001). Similarly, a higher rate of Boer kid than local breed kid was reported in Ethiopia (Molla, 2016; Tesema, Alemayehu, et al., 2020).

A 1‐kg increase in kid birth weight (KBW) reduces the probability of mortality by 32.5% (*p*‐value < 0.001). Similar to birth weight, higher doe weight was associated with a decreased incidence of mortality. A 1‐kg increase in doe PPW reduces the probability of mortality by 6.4% (*p*‐value < 0.001). In line with our current findings, KBW has a significant effect on the survival rate of kids and lambs in most reports. As the birth weight of kids and lambs increases, the mortality rate will be reduced significantly (Chauhan et al., 2019; Getachew et al., 2015; Husain et al., 1995; Tesema, Deribe, et al., 2020). Chauhan et al. (2019) reported that a 1‐kg increase in KBW decreased the risk of death by 78%; similar to KBW, a 1‐kg increase in doe weight was also associated with a 2% decrease in the risk of kid death. Colostrum and milk production are suboptimal in low‐weight dams, resulting in starvation and poor immunity of kids. Low doe weight is also associated with reduced birth weight, which is related to an increased incidence of mortality. Due to undernutrition, the maternal care and recognition abilities of the mother are compromised, resulting in a poor bond between mother and kid (Chauhan et al., 2019; Dwyer et al., 2016).

Single born kids had a better survival rate than twins in this study. Twins birth kids were 1.512 times higher risk of mortality rate (*p*‐value = 0.001) than single born kids. Similarly, twin birth kids and lambs are at higher risk of mortality in most reports (Husain et al., 1995; Getachew et al., 2015; Chauhan et al., 2019; Tesema, Deribe, et al., 2020).

In addition to animal‐related factors, mortality is affected by environmental factors such as the season of birth, average precipitation and year of birth. Kids born during the years 2012/2013, 2015/2016, 2016/2017 and 2017/2018 were at higher risk of mortality (*p* < 0.05) than the base 2011/2012 birth year. Generally, with each passing year, there has been an increase in the hazard of kid mortality. This may be due to the increased flock size from time to time on the farm, resulting in a shortage of browsing land and difficulties in management. Other reasons can be due to fluctuations in flock management, climatic variables, disease incidence and parasite infestation throughout the year.

A 1 ml increment of 15 days of average precipitation significantly reduced kid mortality by 7.8% (1–0.922, *p*‐value < 0.001). In this research area, natural feed resources for goats, such as shrubs, trees and grass, are highly dependent on the availability of precipitation. Thus, kid mortality will be reduced when the area receives higher precipitation, particularly during the dry and short rainy seasons. Kids born during the long rainy season were also at higher risk of mortality (*p*‐value = 0.008) than those born during the dry season. The better survival rate of kids born in the dry season contradicts other season effects on kid survival in India (Husain et al., 1995) and lamb survival in Ethiopia (Getachew et al., 2015), who reported that kids and lambs born during the dry season have a lower survival rate. Seasons have no significant effect on Dorper cross local lambs (Tesema, Deribe, et al., 2020), and lambs born during the dry season are at higher risk than any other seasons (Getachew et al., 2015). A better kid survival rate was reported for kids born during rainy (July to October) (Husain et al., 1995) and summer and rainy (March to June and July to October, respectively) (Chauhan et al., 2019) in Indian climatic conditions. This is most likely due to breed and climate zone differences in these studies. The lower mortality rate of kids that born during the dry season of Ataye (Efratana Gidim district) research site, may be due to goats better access to natural feed sources such as grass, shrub and tree plants in this season. The biomass of natural feeds such as trees and shrubs is highest during subsequent months (dry season) after the end of the main rainy season in the area.

The mortality risk of kids in their first week of age was highest, whereas it was lowest in their older ages (180–2431 days of age), which agreed with most studies (Chauhan et al., 2019; Getachew et al., 2015, p. 2; Husain et al., 1995; Tesema, Deribe, et al., 2020; Tesema, Alemayehu, et al., 2020) (Table 4).

**TABLE 4:** Piecewise exponential model analysis of the effect of explanatory variables on kid mortality at the Ataye Boer centre

Risk factors		HR	HR 95% CI		*p*‐Value
Breed	CHG cross Boer	1			
	Pure Boer	2.505	1.707	3.675	<0.001
Sex	Female	1 (base)			
	Male	1.184	0.977	1.435	0.086
Kid BWT		0.675	0.571	0.797	<0.001
Doe PPWT		0.956	0.936	0.976	<0.001
Parity	1st	1.074	0.753	1.533	0.693
	2nd	1.02	0.718	1.449	0.913
	3rd	1.265	0.877	1.826	0.209
	4th	1 (base)			
	5th	1.203	0.778	1.859	0.407
	6th	0.961	0.467	1.976	0.913
Birth type	Single	1 (base)			
	Twin	1.512	1.192	1.917	0.001
Birth year	2011/2012	1 (base)			
	2012/2013	2.541	1.512	4.269	<0.001
	2013/2014	1.511	0.955	2.392	0.078
	2014/2015	1.371	0.865	2.174	0.179
	2015/2016	2.209	1.371	3.558	0.001
	2016/2017	2.868	1.7	4.838	<0.001
	2017/2018	4.124	2.398	7.093	<0.001
	2018/2019	1.284	0.658	2.502	0.463
Kidding season	Dry	1 (base)			
	Short rain	1.288	0.973	1.705	0.077
	Long rain	1.863	1.172	2.962	0.008
Precipitation		0.922	0.882	0.964	<0.001
Time interval (days)	0–7	68.717	54	87.444	<0.001
	7–90	5.485	4.201	7.161	<0.001
	90–180	2.162	1.491	3.136	<0.001
	180–2431	1 (base)			

Abbreviations; BWT, birth weight; CHG, Central Highland goat; HR, Hazard Ratio; PPWT, post‐partum weight.

Constant piecewise exponential proportional hazard regression analysis of risk factors indicates that breed and the year of goat entrance to the farm were associated with (*p*‐value < 0.05) adult goat mortality rate. However, sex was not associated (*p*‐value > 0.05) with the goat mortality rate. Boer goats compared with CHG goats were 1.503 times at higher mortality (*p*‐value = 0.02). In line with our result, a poor survival rate (poor doe longevity) of Boer goats compared with Kiko and Spanish goats was reported (Pellerin & Browning, 2012). Goats that joined the farm in 2017 were 7.083 times at a higher rate of mortality than goats that joined in 2011 (*p*‐value < 0.001) (Table 5). Mortality variation across the year is due to fluctuations in flock management, climatic variables, disease incidence and parasite infestation throughout the year. A high incidence of acute diarrhoea was clinically and serologically diagnosed as PPR‐related mortality was recorded in goats that joined in 2017 (Alemnew, Asfaw, et al., 2020). This may be due to the low immunity of the newly introduced goats to PPR, while the parent stock in the farm was annually vaccinated against PPR.

**TABLE 5:** Constant piecewise exponential proportional hazard regression analysis of the effect of explanatory variables on adult goat mortality at the Ataye Boer centre

Variables	Class	HR	95% CI	*p*‐Value
Breed	CHG	Base		
	Boer	1.503	1.067–2.118	0.02
Sex	Male	1		
	Female	1.434	0.84–2.449	0.186
Year	2011	1		
	2013	1.307	0.877–1.946	0.188
	2017	7.083	4.661–10.763	<0.001
Time interval (months)	0–6	1.352	0.982–1.861	0.065
	6–9	0.219	0.089–0.541	0.001
	9–18	0.518	0.346–0.775	0.001
	18–24	1.29	0.88–1.889	0.192
	24–94.06	1		

Abbreviations; CI, confidence interval; CHG, Central Highland goat; HR, Hazard Ratio.

## 4 CONCLUSIONS

The kid and adult goat mortality rates in the present study at Ataye Boer goat evaluation research site were higher than those in other reports in Ethiopia and abroad. The kid mortality rate was influenced by both animal‐ and environmental‐related factors, which is in line with previous studies. Management practices aimed at improving the health and survival of goats need to focus on countering unfavourable factors. Doe giving birth during the long rain season and low precipitation, kids in their first week of age, light, and twin kids and kids born from light does should receive special attention. Similarly, goats newly introduced to farms need adequate care until they adapt to the environment. Fleshing of does during the early meeting to improve the post‐partum weight of does and kids is also important to reduce both kid and doe mortality at and after kidding. Immunisation of newly introduced kids to common endemic diseases in the area, extensive control of ticks to break down heartwater transmission and use of proper comfortable housing to reduce the stress of goats are recommended to minimise mortality. Scheduling a mating programme (May to early July) to match the kidding period to the less kid mortality seasons (end of the long rainy season of the area) of flocks is also important.

## CONFLICT OF INTEREST

The authors declare no conflict of interest.

## ETHICS STATEMENT

The authors confirm that the ethical policies of the journal, as noted on the journal’s author guidelines page, have been adhered to. The research were reviewed and approved at the Amhara Regional Research Institute annual agricultural research review forum (Code: Ls/Ab/Sh15/ DB‐2010/14).

## AUTHOR CONTRIBUTIONS

**Erdachew Yitagesu**: Conceptualisation, data curation, formal analysis, investigation, methodology, resources, software, supervision, validation, visualisation, writing—original draft, and writing—review and editing. **Enyiew Alemnew**: Conceptualisation, data curation, resources, supervision and validation.

### PEER REVIEW

The peer review history for this article is available at https://publons.com/publon/10.1002/vms3.876.

## Data Availability

None.
